# Effect of Dynamic Balance Exercises Based on Visual Feedback on Physical Function, Balance Ability, and Depression in Women after Bilateral Total Knee Arthroplasty: A Randomized Controlled Trial

**DOI:** 10.3390/ijerph17093203

**Published:** 2020-05-05

**Authors:** Ju-Yeon Lee, Jung-Hee Kim, Byoung-Hee Lee

**Affiliations:** 1 Graduate School of Physical Therapy, Sahmyook University, Seoul 01795, Korea; ljyljy1115@naver.com; 2 Department of Physical Therapy, Andong Science College, Andong 36616, Korea; mirrorneuron98@gmail.com; 3 Department of Physical Therapy, Sahmyook University, Seoul 01795, Korea

**Keywords:** total knee arthroplasty, visual feedback, balance exercise, women

## Abstract

The aim of this study was to compare the effects of dynamic balance exercises with and without visual feedback on recovery from total knee arthroplasty. The participants were 30 women who underwent total knee arthroplasty more than one week before the study, and were randomly allocated into two groups. The average ages of the experimental and control groups were 70.13 and 69.00 years, respectively. The dynamic balance exercise with visual feedback (VF) group practiced dynamic balance exercises using a laser pointer for five 30-min sessions over a 4-week period. The dynamic balance exercise without visual feedback (control) group practiced dynamic balance exercises for five 30 min sessions over a 4 week period. The following clinical measures were used for assessing physical function, balance ability, and depression. Compared with the control group, the VF group showed significant improvements in the physical performance test, Western Ontario and McMaster Universities Arthritis Index (WOMAC), confidence ellipse area (CEA), path length (PL), average velocity (AV), and Timed Up and Go test (TUG test) (*p* < 0.05). Furthermore, the VF group showed significant improvements in all post-surgery outcome measures compared with the pre-surgery values (*p* < 0.05). The above results indicated that the dynamic balance exercises based on visual feedback improved physical function and balance ability in patients following total knee arthroplasty, suggesting the need for effective rehabilitation programs for patients with total knee arthroplasty.

## 1. Introduction

Total knee arthroplasty (TKA) reduces symptoms associated with degenerative knee osteoarthritis, improves daily activities, and reduces the risk of falling [1,2]. Symptoms of late degenerative knee osteoarthritis include severe knee pain, joint stiffness, limited range of motion (ROM), impaired function and inherent water-soluble impairment, and an increased risk of falling [3]. Traditional exercise therapy for TKA includes knee joint ROM and lower extremity strength exercises. Traditional exercise therapy, however, has been reported to limit the ROM of the knee joint, thereby increasing knee joint pain and retaining residual intrinsic soluble sensory phenomena [4].

Previous studies have reported the effects of improving the balance ability of patients following TKA by applying traditional exercise therapy and balance exercises [5]. In a previous study, laser pointers worn around the knee joints provided external visual feedback regarding posture during knee flexion and squeezing in adults with large Q-angles. In addition, a reduction in knee joint pain and an increase in balance ability have been reported in patients with total knee replacement following visual feedback-based balance exercises [6].

Visual information is advantageous because sensory information of the external environment can easily be recognized and directly collected compared with intrinsic water-soluble sensations [7]. Although research has been conducted on effective exercise therapies following TKA, no studies have determined the effect of dynamic balance exercises on physical function, balance ability, and depression of TKA with or without visual feedback using a laser pointer. The purpose of this study was to investigate the effect of visual feedback-based dynamic balance exercises using a laser pointer on the treatment process of patients with bilateral total knee replacement (TKR).

## 2. Materials and Methods

### 2.1. Methods

Thirty female patients who underwent bilateral TKA in Bundang B Hospital (Seongnam, Gyeonggi Province of Korea) participated in the study. All patients agreed to participate in this experiment after being informed of the progress, purpose, and possible side effects of the experiment. The inclusion criteria of the study were: patients who underwent bilateral total knee replacement for degenerative knee osteoarthritis more than one week before the study start date, those who had no visual impairments or deficiencies, and who could understand and follow the researcher’s instructions. The exclusion criteria were participants who had a history of knee surgery due to other causes within 6 months, underwent TKA as a result of other diseases such as purulent or rheumatoid arthritis, underwent unilateral TKA, or were older than 80 years. Patients with unilateral TKA could also apply the intervention used in this experiment, but due to the compensatory activity involving the healthy lower extremities, it may have been difficult to clearly assess the differences in effectiveness of the intervention. Participants indicated their consent for participation in the study and were randomly assigned to an AR-based exercise group or control group through a random draw. All participants picked a piece of paper with a number written in black or white from a box containing 30 pieces of paper. This study was conducted with the approval of the Research Institutional Review Board of Sahmyook University (approved number: 2-7001793-AB-N-012018125HR).

### 2.2. Experimental Group

The experimental group underwent visual feedback-based dynamic balance exercise sessions which were 30 minutes long using a laser pointer five times a week for 4 weeks, and conventional physical therapy was performed for five 30 min sessions a week for 4 weeks.

**Figure 1:**
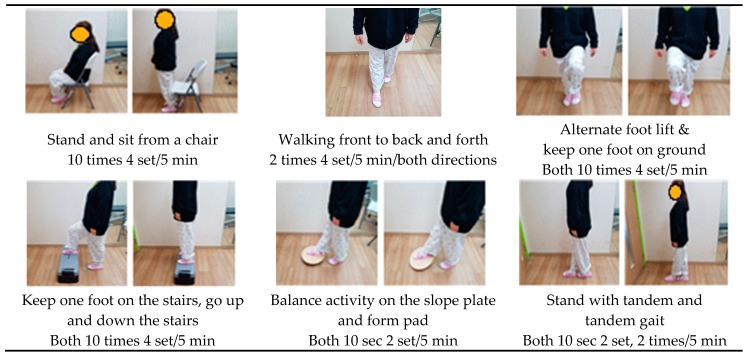
Visual feedback-based dynamic balance exercise program.

A belt fitted with a laser pointer was placed 8 cm above the kneecap of the participant, and the angle of the laser pointer device was adjusted so that the position of the laser beam was 3 cm in front of the second toe on the instep. The therapist adjusted the position of the laser pointer so that the knee was positioned over the participant’s toes, considering the length of the tibia and bending and ROM of the knee. Visual feedback-based dynamic balance using a laser pointer was applied to one knee first for 15 min, and then to the other knee [8]. The visual feedback-based dynamic balance exercise program is shown in Figure 1.

Conventional physical therapy includes warm-up and knee mobility exercises, ankle pull and push exercises, isometric exercises for knee flexion and elbow muscles, hip isolation and voluntary muscle tone strengthening, squatting against a wall, and muscle strengthening. Exercises were performed for 30 min, 5 times a week for 4 weeks. Continuous passive motion was performed for 60 min, 5 times a week for 4 weeks (Table 1) [9].

**Figure 2:**
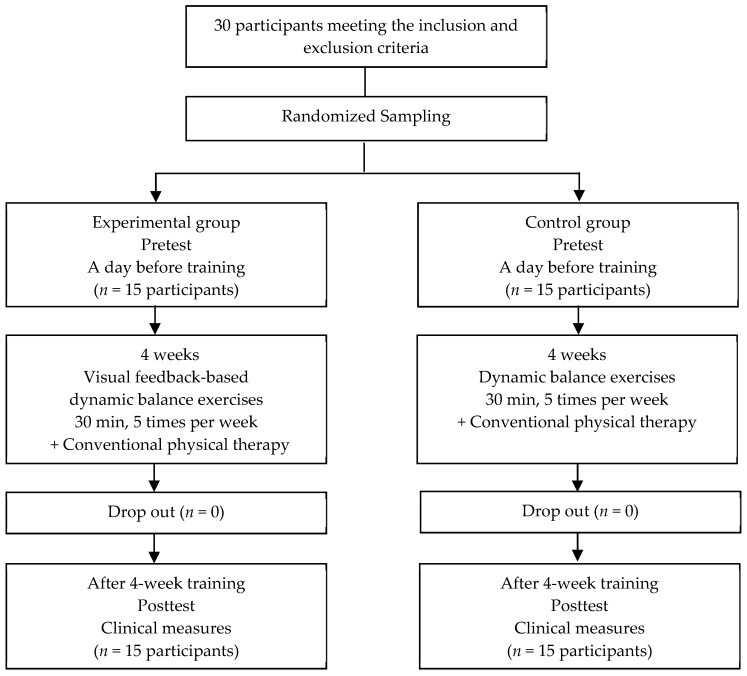
Flow chart of participants through the training program.

For the control group, dynamic balance exercises without visual feedback and conventional physical therapy were performed for 30 min, 5 times a week for 4 weeks. The dynamic balance training program conducted by the control group consisted of the same program as that performed by the experimental group and except for the use of the visual feedback device. Continuous manual exercise was performed for 60 min, 5 times per week for 4 weeks. The progress of this study is shown in the following chart (Figure 2).

### 2.3. Outcome Measures

An electronic goniometer (Biometrics, Ladysmith, VA, USA, 2008) was used to evaluate the ROM of the knee joint flexion. In the supine position, one side of the goniometer was positioned to face the femur head and the other side was pointed towards the lateral malleolus of the ankle to measure the angle of bending during active bending (ICC = 0.89). The measurement was performed three times in total, and the average value was used for statistical analysis [10].

The Western Ontario and McMaster Universities Arthritis Index (WOMAC) is a self-assessment questionnaire that assesses pain, activity of daily living, functional mobility, quality of life, gait, and physical function. It consists of 5, 2, and 17 items of pain, stiffness, and physical function, respectively [11]. From a total achievable score of 96 points, a lower score indicates less severe symptoms or physical disability. An electronic pressure pain gauge (PainTest™ FPX 25 Algometer; Wagner instrument, Greenwich, CT, USA, 2015) was used to assess the pain level in patients with TKA [12]. The knee was bent as much as possible in the side-lying position, and pain was measured three times at the 1–2 cm point of the femoral tuberosity inside the knee joint. An increase in value indicated lower pain levels [13].

A force plate (FDM-L Multifunction Force Measuring Plate; Zebris Medical, Isny, Germany, 2016) was used to measure static balance ability. Participants stood directly on the force plate for 20 seconds, looking at a fixed point ahead, while the 95% confidence ellipse area (CEA), center of pressure (COP), path length (PL), and COP average velocity (AV) were measured. The intraclass correlation coefficient (ICC) of the force plate was 0.99 [14]. The Timed Up and Go test (TUG) was used to measure dynamic balance. This test measures the time taken to get up from a chair without armrests, walk 3 m across the floor, and back again before returning to the sitting position. If the test result was <10 seconds, there was no risk of falling, and if >30 seconds, there was a high risk of falling, requiring help in daily activities [15]. The ICC of the TUG test was 0.97 [16].

A hospital anxiety and depression evaluation (HADS) was performed to evaluate the degree of depression. HADS is a reliable questionnaire (ICC = 0.84) used to assess anxiety and depression [17]. Each item has a four-point scale (range 0–3 points), with a maximum score of 21. Higher scores indicated higher levels of anxiety or depression [18].

### 2.4. Statistical Analysis

Statistical analysis was performed using SPSS ver. 18.0 (SPSS Inc., Chicago, IL, USA). Data are presented as the mean and standard deviation. A Shapiro–Wilk normality test was performed, and all items were normally distributed. General characteristics of participants are presented as descriptive statistics. Independent t-tests were used to compare differences between the groups. A paired t-test was used to compare differences in the groups. The significance level for all data was set at 0.05.

## 3. Results

The experimental results showed that all items were homogeneous in the experimental and control groups (Table 2). The overall effect size index for all outcome measures and power of the study were 0.53. To minimize type II errors (power of 80%), 15 patients were required.

### 3.1. Range of Motion (ROM)

There was a significant difference between the experimental and control groups with respect to the ROM of the knee joint flexion (*p* < 0.001), but there was no difference between the groups (Table 3). There was a significant increase in the pain threshold of the right knee joint in the experimental and control groups, and there was a significant difference in the experimental group than the control group (*p* < 0.01). The experimental group showed a significant increase before and after training in the pain threshold of the left knee joint (*p* < 0.001), and there was no significant difference in the control group. Comparisons between the groups indicated that the experimental group showed a more significant difference than the control group (*p* < 0.05).

### 3.2. Pain

Regarding the WOMAC assessment of pain, the experimental and control groups showed a significant difference (*p* < 0.001) (Table 4). In the comparison between groups, the experimental group showed a significant increase compared with the control group (*p* < 0.05). In the WOMAC assessment of stiffness, the experimental and control groups showed a significant increase (*p* < 0.05), and in the comparison between groups, the experimental group showed a significant increase compared with the control group (*p* < 0.05). In the WOMAC assessment of body function, the experimental and control groups showed a significant difference (*p* < 0.001), and in the comparison between groups, the experimental group showed a significant increase compared with the control group (*p* < 0.05).

### 3.3. Balance

#### 3.3.1. Static Balance

In the evaluation of CEA, the experimental group showed a significant improvement before and after training (*p* < 0.001), but there was no significant improvement in the control group (Table 5). There was a significant difference in the experimental group compared with the control group in the effect comparison (*p* < 0.01). In PL, the experimental group showed a significant improvement before and after training (*p* < 0.01), but there was no significant improvement in the control group. In the comparison between groups, the experimental group showed a significant difference compared to the control group (*p* < 0.05). Regarding AV, the experimental group showed a significant improvement before and after training (*p* < 0.01), whereas the control group did not. In the comparison between groups, the experimental group showed a significant difference compared with the control group (*p* < 0.05).

#### 3.3.2. Dynamic Balance

In the TUG test, the experimental and control groups showed a significant difference before and after training (*p* < 0.001), but the comparison between groups showed a significant difference in the experimental group (*p* < 0.01) (Table 5).

### 3.4. Anxiety and Depression

There was a significant difference in the experimental and control groups pre- and post-training in HADS anxiety, but that there was no significant difference between groups (Table 6). There was a significant difference in the experimental and control groups in HADS depression before and after training (*p* < 0.01), but there was no significant difference between groups.

## 4. Discussion

The recovery of the knee flexion angle after TKA is important for the prevention of joint fibrosis complications, in which the knee joint does not bend but is instead stuck, making walking difficult [19]. Contraction of the hamstring muscle is essential to increase the active bending angle of the knee joint [20]. In this study, visual feedback was provided, including active flexion and extension of the knee joint; walking forwards, backwards, left, and right; keeping one foot on the stairs, and moving up and down the stairs.

### 4.1. Range of Motion (ROM)

In this study, both the experimental and control groups showed a significant increase in ROM (*p* < 0.001), but there was no significant difference between groups. Huber et al. [21] made 45 patients with TKA undergo balance and proprioceptive sensory exercises for 6–12 weeks. The knee ROM in the experimental group ranged from 115.7° to 113.1° after 3 months, but there was no significant difference between groups. In this study, the experimental group repeatedly trained the flexion of the knee joint close to the sagittal plane based on visual information presented by the laser pointer. As a result, hamstring muscle activity and knee flexion control in daily living improved. The patient was maintained in a supine position for measuring the knee flexion angle. Differences between the posture in training situations and that in evaluation are considered causes of insufficient induction of the improved hamstring activity.

### 4.2. Pain

In the evaluation of pressure pain in this study, pain in the experimental group increased from 2.63 kg/cm^2^ to 3.86 kg/cm^2^ and 3.75 kg/cm^2^ to 2.83 kg/cm^2^ on the right and left sides, respectively (*p* < 0.05). The experimental group showed a significant difference compared with the control group (*p* < 0.05). Unexpected motion of the knee joint on an unstable surface caused the laser pointer to move beyond the reference point, and the participant induced quadricep muscle activity to correct it. It is important to avoid additional pain due to harmful mechanical stimulation of the knee during dynamic balance exercises. The visual feedback-based exercises using laser pointers helps to reduce pain by reducing mechanical friction by re-learning that knee movements should occur on the sagittal plane [22].

After surgery, pain may occur owing to hyperactivity of the pain receptors in the skin or soft tissues, and retraining may fail due to reduced muscle strength around the knee joint and deterioration of joint sensory perception [23]. In this study, the pain score of the WOMAC decreased from 13.87 to 7.60, the stiffness score decreased from 5.80 to 3.13, and the physical function score decreased from 53.73 to 30.00 (*p* < 0.05).

Jogi et al. [5] applied dynamic balance exercises to patients with TKA, and the WOMAC physical function score decreased from 37 to 14. Park et al. [24] reported that physical therapy with motion observation training performed in patients with TKA resulted in the reduction of the WOMAC stiffness score from 7.56 to 3.22 and WOMAC body function score from 76.11 to 21.67. The difference was significantly lower than that in the control group.

The visual feedback-based exercise program of this study aimed to prevent abnormal weight load and guide-stable knee joint alignment due to the visual criteria presented by the laser pointer. This induced improvements in physical function, including knee pain and joint stiffness, by inducing improvements in the motion of the knee joint, prevention of asymmetric joint alignment, and reduction of mechanical friction of the knee [22,23].

### 4.3. Balance

In this study, the CEA of the experimental group decreased from 485.93 mm^2^ to 274.82 mm^2^, PL decreased from 361.16 mm to 265.69 mm, and AV decreased from 12.80 mm/s to 9.91 mm/s. The experimental group showed a significant difference compared with the control group (*p* < 0.05). In a study of patients with TKA (*n* = 14) or total hip arthroplasty (*n* = 13), Jogi et al. [25] showed that the CEA measured by the Advanced Mechanical Technology Inc. (AMTI) pressure plate (Advanced Mechanical Technology Inc., Newton, MA, USA) after 14 weeks was 100 mm^2^ in the balance exercise group and 270 mm^2^ in the control group.

To maintain constant balance in the upright position, coordination of the muscles around the hip and knee joints is required. In this study, participants in the experimental group were instructed to move as closely as possible to the path of the laser pointer beam. This condition may induce activity of the biceps femoris, psoas major, sartorius, and iliacus, which are necessary for the regulation of static balance, and they increase static balance by minimizing unnecessary movements in the hip [26]. The experimental group showed a significant improvement compared with the control group, decreasing from 26.08 sec before the experiment to 11.89 sec after the experiment in the TUG test (*p* < 0.01). The visual feedback-based dynamic balance training in this study consisted of motions similar to those in the TUG test, such as standing, tandem gait, getting up, and sitting in a chair. The results of repetitive training of these movements indicated the promotion of dynamic balance [15]. In addition, it is thought that the improvement of the dynamic balance ability was induced through balance training on a narrow base surface, such as walking based on visual feedback [27].

### 4.4. Anxiety and Depression

In the evaluation of anxiety and depression using HADS, the post-test values of the experimental and control groups were significantly improved, but the experimental group showed no significant difference in anxiety and depression compared with the control group. The results of HADS in this study were difficult to interpret with regard to whether the patient’s anxiety and depression were due to a decrease in physical function, change in psychological state due to environmental changes, or personal problems. However, both the experimental and control groups showed a significant decrease in anxiety and depression, indicating a positive effect. Since the level of mental stability of patients before and after surgery affects the level of motivation and social participation after surgery, the application of physical or mental training programs that can lead to improvements in negative psychological status should be considered.

The limitations of this study are as follows. First, generalization of the research results is difficult owing to the small number of participants. Since follow-ups were not conducted in this study, it is difficult to confirm sustained treatment effects. The participants in this study were patients who visited Bundang B Hospital. Therefore, it is difficult to generalize the results to all TKA patients.

## 5. Conclusions

Through this study, visual feedback-based dynamic balance exercises were found to be effective in improving body function, balance ability, and depression in TKA patients post-operation. Visual feedback-based balance training can induce higher levels of patient participation in the rehabilitation process of TKA than general physical therapy, and it is considered an effective method in improving physical function and movement in everyday life. Therefore, we suggest visual feedback-based dynamic balance exercises as an effective intervention method for patients undergoing total knee replacement.

## Figures and Tables

**Table 1:** Conventional physical therapy program.

Exercise Program	Time (Duration)
Stretching of the knee flexor and extensor muscle	Both 30 sec, 3 set, 5 min
Mobility training of the Knee flexor and extensor muscle	Both 10 times, 3 set, 3 min
Pulling and pushing of ankle joint	Both 10 times, 3 set, 2 min
Isometric exercise of the knee flexors	10 times, 3 set, 5 min
Isometric exercise of the knee flexor and extensor muscle	10 times, 3 set, 5 min
Isometric exercise of the hip abductor and adductor muscle	10 times, 3 set, 5 min
Bend and stretch knees against the wall	10 times, 2 set, 5 min

**Table 2:** General characteristics of participants (*n* = 30).

Characteristics	Experimental Group (*n* = 15)	Control Group (*n* = 15)	t	*p*/x^2^
Height (cm)	152.93 ± 5.33	155.29 ± 3.25	0.965	0.401
Weight (kg)	56.80 ± 4.66	62.07 ± 10.42	0.954	0.212
Ages (years)	70.13 ± 4.70	69.00 ± 6.44	0.966	0.437
Body mass Index	24.69 ± 2.65	25.71 ± 4.10	0.968	0.489
On set duration (day)	14.13 ± 2.59	13.20 ± 3.57	0.955	0.235

Mean ± SD

**Table 3:** Comparison of body function (*n* = 30).

Body Function	Experimental Group (*n* = 15)	Control Group (*n* = 15)	t	*p*
ROM Rt (°)				
pretest	85.38 ± 22.42	79.05 ± 15.36		
posttest	122.24 ± 11.60	120.01 ± 12.01		
Pre-post	36.86 ± 19.59	40.96 ± 18.53	−0.590	0.560
*t(p)*	−7.288 (0.000)	−8.560 (0.000)		
ROM Lt (°)				
pretest	84.25 ± 20.54	78.75 ± 16.14		
posttest	128.79 ± 8.91	119.04 ± 12.63		
Pre-post	44.53 ± 20.40	40.29 ± 20.11	0.574	0.570
*t(p)*	−8.453 (0.000)	−7.759 (0.000)		
PPT Rt (*kg/cm^2^*)				
pretest	2.63 ± 0.54	2.88 ± 0.94		
posttest	3.74 ± 0.62	3.29 ± 0.75		
Pre-post	1.11 ± 0.47	0.40 ± 0.67	3.318	0.003
*t(p)*	−9.082 (0.000)	−2.307 (0.037)		
PPT Lt (*kg/cm^2^*)				
pretest	2.75 ± 0.49	2.69 ± 0.72		
posttest	3.83 ± 0.58	2.99 ± 0.61		
Pre-post	1.08 ± 0.59	0.31 ± 1.05	2.489	0.019
*t(p)*	−7.077 (0.000)	−1.141 (0.273)		

*p* < 0.05, Mean ± SD; ROM = range of motion; Rt= right side; Lt = left side; PPT = pressure pain threshold.

**Table 4:** Comparison of the Western Ontario and McMaster universities osteoarthritis index (WOMAC) scores (*n* = 30).

WOMAC Index	Experimental Group (*n* = 15)	Control Group (*n* = 15)	t	*p*
WOMAC Pain				
pretest	138.87 ± 4.07	12.13 ± 2.67		
posttest	7.00 ± 3.68	8.53 ± 3.14		
Pre-post	6.87 ± 4.29	3.60 ± 2.53	2.540	0.017
*t (p)*	6.198 (0.000)	5.511 (0.000)		
WOMAC Stiffness				
pretest	5.80 ± 1.42	5.07 ± 1.10		
posttest	3.13 ± 1.60	3.80 ± 1.66		
Pre-post	2.67 ± 1.11	1.27 ± 1.67	2.705	0.012
*t (p)*	9.282 (0.000)	2.942 (0.011)		
WOMAC Physical function				
pretest	53.73 (6.64)	49.33 ± 6.44		
posttest	30.00 ± 9.96	34.67 ± 13.03		
Pre-post	23.73 ± 7.73	14.67 ± 12.20	2.431	0.022
*t (p)*	11.888 (0.000)	4.657 (0.000)		

*p* < 0.05, Mean ± SD; WOMAC = the Western Ontario and McMaster universities osteoarthritis index.

**Table 5:** Comparison of balance test (*n* = 30).

Balance	Experimental Group (*n* = 15)	Control Group (*n* = 15)	t	*p*
CEA				
pretest	485.93 ± 93.98	417.71 ± 107.65		
posttest	274.82 ± 157.38	368.49 ± 125.18		
Pre-post	211.11 ± 154.37	49.22 ± 105.37	3.355	0.002
*t (p)*	5.296 (0.000)	1.809 (0.092)		
PL				
pretest	361.16 ± 59.23	305.51 ± 116.36		
posttest	265.69 ± 102.63	303.24 ± 73.20		
Pre-post	95.47 ± 115.47	2.27 ± 113.56	2.229	0.034
*t (p)*	3.202 (0.006)	0.077 (0.939)		
AV				
pretest	12.80 ± 2.31	10.82 ± 4.00		
posttest	9.91 ± 2.48	11.20 ± 2.71		
Pre-post	2.89 ± 3.61	−0.38 ± 3.56	2.499	0.019
*t (p)*	3.106 (0.008)	−0.411 (0.687)		
TUG				
pretest	26.08 ± 12.19	24.84 ± 7.81		
posttest	11.89 ± 2.50	19.66 ± 5.89		
Pre-post	14.19 ± 10.78	5.17 ± 4.22	3.016	0.007
*t (p)*	5.096 (0.000)	4.752 (0.000)		

*p* < 0.05, Mean ± SD; CEA = 95% confidence ellipse area; PL = COP path length; AV = COP average velocity; TUG = time up and go test.

**Table 6:** The changes in the hospital anxiety and depression scale (HADS) (*n* = 30).

HADS	Experimental Group (*n* = 15)	Control Group (*n* = 15)	t	*p*
HADS anxiety				
pretest	9.13 ± 5.07	8.40 ± 4.27		
posttest	5.00 ± 3.63	4.33 ± 3.92		
Pre-post	4.13 ± 5.40	4.07 ± 3.28	0.041	0.968
*t (p)*	2.966 (0.010)	4.797 (0.000)		
HADS depression				
pretest	10.47 ± 2.97	9.60 ± 1.76		
posttest	5.53 ± 2.59	4.20 ± 2.60		
Pre-post	4.93 ± 4.54	5.40 ± 2.67	−0.343	0.735
*t (p)*	4.206 (0.001)	7.841 (0.000)		

*p* < 0.05, Mean ± SD; HADS = Hospital anxiety and depression scale.
